# Protectiveness of Artesunate Given Prior Ischemic Cerebral Infarction Is Mediated by Increased Autophagy

**DOI:** 10.3389/fneur.2018.00634

**Published:** 2018-08-17

**Authors:** Ming Shao, Yue Shen, Hongjing Sun, Delong Meng, Wei Huo, Xu Qi

**Affiliations:** ^1^Department of Orthopedics, The First Affiliated Hospital of Harbin Medical University, Harbin, China; ^2^Department of Neurology, The First Affiliated Hospital of Harbin Medical University, Harbin, China

**Keywords:** mTOR, autophagy, artesunate, infarction, Ischemic cerebral infarction

## Abstract

**Background:** Ischemic cerebral infarction is a severe clinical condition that can cause serious mortality. Artesunate, an anti-malarial drug that is widely used in cancer treatment, is known to facilitate accelerated cell apoptosis. The aim of this study is to explore the possible neuroprotective effects of artesunate on hypoxic-ischemic cells in rats.

**Methods:** Middle cerebral artery occlusion (MCAO) rats were treated with artesunate in different doses to observe their survival rate. Primary hippocampal neurons were deprived of oxygen-glucose to induce ischemia symptoms. Western blot was performed to determine the protein expressions of p-mTOR, Beclin-1, and Mcl-1. A five-point scale was used to detect neurological deficit. Cell apoptosis was measured using a TUNEL assay.

**Results:** Artesunate supplementation protected MCAO rats from death and ameliorated brain injury among them. Artesunate administration decreased the expression of p-mTOR, increased the expressions of Beclin-1 and Mcl-1, and decreased the activity of caspase-3 in both the rats' ischemia cerebral cortices and their primary ischemia hippocampal neurons when compared with artesunate-absent ischemic brains and cells. The neuroprotective effects of artesunate were abolished by either leucine (LEU) or 3-MA, while the effects of rapamycin were reversed by 3-MA. *In vivo* experiments verified the protective effects of artesunate on brain-infarct rats.

**Conclusion:** The results indicate the protectiveness of artesunate against ischemic cerebral infarction, whereas the protectiveness might increase autophagy through regulating the activity of mTOR.

## Introduction

Cerebral infarction, also known as stroke, is the second leading cause of disability and death worldwide (1). Every year, ~15 million people are diagnosed with a cerebral infarction, while about 5 million people die from the disease globally. Even though progress has been made on fundamental research and clinical treatment, the patients who suffer from cerebral infarction are still unsatisfied. Traditionally, cerebral infarction is thought to be caused by insufficient cerebral blood flow, while oxidative stress and inflammation also play an important role (2–4). Ischemic brain infarcts are a type of cerebral infarction that not only affect the middle cerebral artery, but also induce early mortality (5). Nevertheless, the indistinct mechanism or the clinical method limit the therapy for patients with cerebral infarction.

Artesunate is a traditional anti-malarial Chinese drug that has been proven to play an important role in cancer treatment due to its role in inducing cell apoptosis (5, 6). Studies have suggested that oxidization and inflammation have a vital role in cell death induced by artesunate; for example, artesunate enhances cell apoptosis by stimulating anticancer toxicity (7, 8). Moreover, artesunate induces severer mitochondrial injury that is mediated by ROS (9). A previous study has proven that artesunate has an effect on the therapy of cerebral malaria during its acute phase (10), but whether artesunate affects cerebral infarction is still unclear.

Autophagy is a catabolic process that can damage organelles and specific proteins. The activation of the mammalian target of rapamycin (mTOR) seems to affect protein translation, cellular response and autophagy. Generally, autophagy is known to eliminate toxins, pathogens, and several modified cytoplasmic, and further protects cells against injury. A previous study reported that activated mTOR in amino acid-rich conditions could inhibit the occurrence of autophagy, while when amino acids are limited extracellularly, autophagy recycles intracellular seem to provide an alternative resource for amino acids (11). Other studies have provided key insights into mTOR as an important factor in autophagy. For example, Nazio et al. found that mTOR inhibits autophagy through regulating ULK1 ubiquitylation (12), while Liang et al. supported mTOR as a therapeutic target by regulating YAP in the tuberous sclerosis complex (13). Zhang et al. suggest that mTOR promotes autophagy and protects rats from osteoarthritis by deleting specific cartilage (14). The mechanism of cerebral infarction has also been studied by many researchers, as well as its autophagy. For example, ebselen was recognized to reduce autophagic activation in ipsilateral thalami with cerebral infarction (15). Other studies have indicated that AKT/mTOR signaling is related to ischemic cerebral infarction. Beclin-1 is an autophagy gene that relates to cell death, Mcl-1 is a member of the anti-apoptotic Bcl-2 family, and caspase-3 plays an important role in cell apoptosis through the activation of death-related proteases. All those genes are favorable to detect cell death.

3-MA is an autophagic inhibitor that can be used to block the formation of autophagosome and autophagic vacuoles (16). Leucine (LEU) is a p-mTOR agonist and has been identified to mediate p-mTOR phosphorylation (17). Rapamycin is an inhibitor of mTOR, and has been widely used in regulating the activity of mTOR-signaling pathways (18).

In this study, 3-MA, LEU, and rapamycin were used to analyze the role of artesunate, and it was found that mTOR was mediated in the autophagy of cerebral infarctions. The aims were to explore the mechanism of mTOR on autophagy and determine whether artesunate affects cerebral infarction by regulating the expression of mTOR and autophagy.

## Materials and methods

### Animals

In this study, a total of 80 Sprague-Dawley (SD) male rats aged 4–6 weeks and weighing 220–250 g each were used. Food and water were provided *ad libitum* in a controlled environment. The study was permitted by the Animal Ethics Committee.

The rats were randomly divided into four groups (*n* = 10 in each group), including Sham (Group 1), middle cerebral artery occlusion (MCAO, Group 2), MCAO+artesunate (30 mg/kg, Group 3) and MCAO+artesunate (60 mg/kg, Group 4). Artesunate was dissolved in the PBS and intraperitoneally injected into the rats 2 h before MCAO. MCAO was performed according to the reference (19). The rats were all under 4% chloral hydrate anesthesia by intraperitoneal injection before the MCAO surgery.

The work was approved by the ethics committee of the First Affiliated Hospital of Harbin Medical University.

### Detection of neurological deficit, cerebral infarct volume, and brain edema

Neurological deficit was detected via motivation ability and visual ability. The five-point scale described previously (20) was used to assess neurological grading; to summarize, “0” presented no apparent deficits; “1” presented left forelimb flexion; “2” presented a decreased grip of the left forelimb while the tail was pulled; “3” presented spontaneous movement in all directions; and “4” presented spontaneous left circling. The experiments were performed by three technicians and the averages of their individual values represented the final results.

Brain edemas were quantified by detecting water content in the rats' brains. After the rats were sacrificed, their brain tissues were taken out and weighed (W1). Then, the tissues were dried and again their weights (W2) were measured. Finally, the water content of the brains was calculated using the formula (W1–W2)/W1 × 100%.

### Western blot

Ischemic cerebrum cells and tissues were lysed using a RIPA Lysis Buffer. Total protein was extracted using a Total Protein Extraction Kit (Takara) according to the manufacturer's instructions, and the quality was detected using the Bradford method. Sodium dodecyl sulfate-polyacrylamide gel electrophoresis (SDS-PAGE) was used to separate the protein extracts. An equal amount of protein was transferred onto a polyvinylidene fluoride membrane, and incubated with primary antibodies (anti-p-mTOR, anti-Beclin-1, anti-Mcl, 1:500, [Sigma, USA]) or β-actin (1:500, [R&D, China]) at 4°C for 24 h. The membranes were then incubated with the secondary antibody (1:1000) for another 2 h at room temperature. The ECL method and Image J software were used to visualize the bands. The antibodies were purchased from Abcam (UK). β-actin acted as an internal control.

### Cell culture and treatment

Primary rat hippocampal neuron that isolated from SD rats. The cells were cultured in a 24 well-plate with 1 × 10^4^/well and cultured with DMEM supplemented with 10% fetal bovine serum at 37°C with 5% CO_2_ in a humidified atmosphere. After 4 h, the medium was replaced by chemically defined medium, and the half of the volume of the medium was replace every 3 days by an equal volume of chemically defined medium.

Oxygen-glucose deprivation treatment was performed as previously described (21). The cells were exposed to a glucose-free solution of RPBM 1640 medium and cultured at 37°C in an incubator with 5% CO_2_ and 95% N_2_ for 2 h.

### Tunel-positive assay

After the cells were treated with artesunate, 3-MA or rapamycin for 2 h and subsequently exposed to gas with oxygen-glucose deprivation for 24 h, TUNEL staining was carried out using a TUNEL Apoptosis Assay Kit (Roche, USA), according to the manufacturer's instructions. The numbers of positive cells were the average value of the ten individual regions of vision.

### Activity of caspase-3

To determine the activity of caspase-3 in rats with different treatments (Sham, MCAO, MCAO+30 mg/kg artesunate and MCAO+60 mg/kg artesunate), the Colorimetric Assay Kit (Genscript, Piscataway, NJ, USA) was used, and the instructions were followed.

### *In vivo* experiments

MCAO rats were randomly divided into four groups, including Group 1: MCAO+PBS, Group 2: MCAO+atesunate (60 mg/kg), Group 3: MCAO+atesunate (60 mg/kg) +3-MA (30 mg/kg), and Group 4: MCAO+3-MA (30 mg/kg). After 24 h, the neurological deficit, cerebral infarct volume and brain edema of the rats in the four groups were measured.

### Statistical analysis

All data were expressed as means±SD. The analysis was performed using SPSS18.0. Statistical differences were processed using one-way analysis of variance (ANOVA) combined with the least significant difference *t*-test independent-sample *t*-test. The post-hoc test was carried out to compare the differences in the individual groups. *P* < 0.05 was recognized as a statistically significant difference.

## Results

### Artesunate protected the cerebrum against infarction

**Figure 1:**
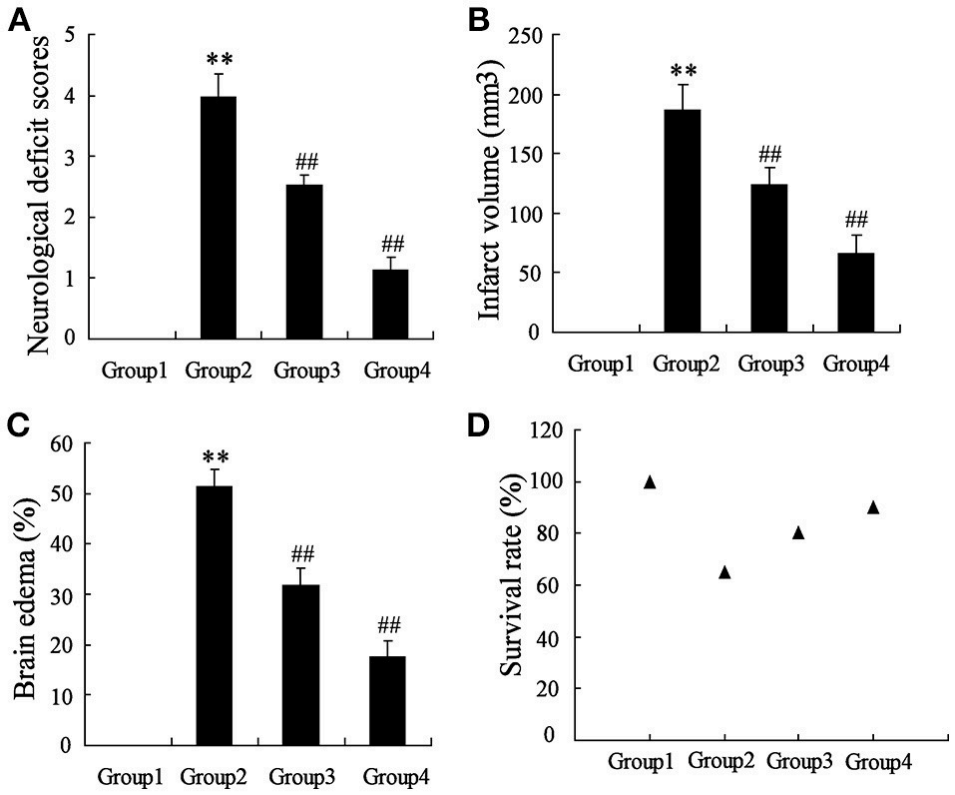
Effects of artesunate on brain injuries. The neurological deficit score **(A)**, infarct volume **(B)**, brain edema **(C)**, and the survival rate **(D)** were significantly increased in MCAO rats, while artesunate reversed the results in a dose-dependent manner. **P < 0.01 vs. Group 1, ^##^*P* < 0.01 vs. Group 2.

To determine the effects of artesunate on cerebral infarction, the rats were divided into four groups as previously described, including Sham (Group 1), middle cerebral artery occlusion (MCAO, Group 2), MCAO+30 mg/kg artesunate (Group 3) and MCAO+60 mg/kg artesunate (Group 4). After 24 h, the mortality rates of Groups 1, 2, 3, and 4 were 0, 35, 20, and 10%, respectively (the data were not shown in the article). Cerebral infarction degrees were detected in the living rats. Compared with Group 1, neurological deficit (Figure 1A), cerebral infarct volume (Figure 1B), and brain edemas (Figure 1C) were significantly higher, while the effects were reversed by artesunate (Groups 3 and 4) based on the dose. In addition, when compared with Group 1, the survival rate was lower in Groups 2, 3, and 4, while the artesunate recovered the survival rate based on dose (Figure 1D). The results revealed that artesunate significantly ameliorates infractions in neurological deficit, cerebral infarct volume and brain edemas based on dose.

**Figure 2:**
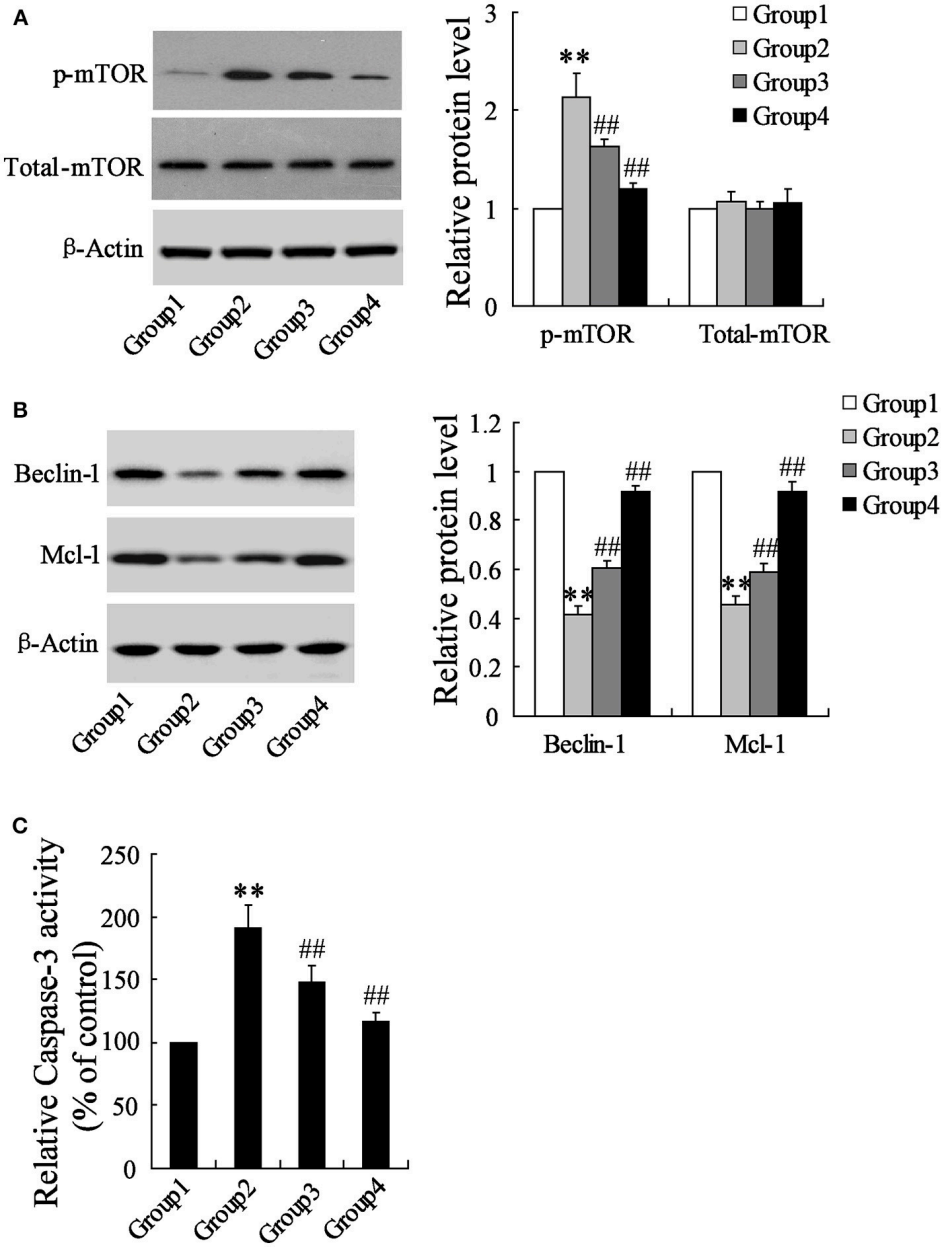
Effects of artesunate on p-mTOR, Beclin-1 and Mcl-1. Ischemia cerebral cortex reflected a significant increase in p-mTOR **(A)**, a significant decrease in Beclin-1 and Mcl-1 **(B)**, and a significant increase of caspase-3 **(C)**, while artesunate supplementation reverses its effects in a dose-dependent manner. **P < 0.01 vs. Group 1, ^##^*P* < 0.01 vs. Group 2.

To detect the effects of artesunate on the cerebral cortex in the ischemic region, a western blot and a caspase-3 activity assay were performed. According to Figure 2A, the protein expression of p-mTOR was prominently higher in Group 2 compared to Group 1, while it was reversed in Group 3 and Group 4 with the dose-dependent artesunate, while the protein expression of total mTOR showed no significant difference. The protein expressions of Beclin-1 and Mcl-1 was lower in Group 2 than in Group 1; however, it was abolished in Groups 3 and 4 with the dose-dependent artesunate (Figure 2B). Furthermore, the activity assay revealed that the activity level of caspase-3 in Group 2 was dramatically higher than Group 1, while its effects were also reversed by artesunate (Groups 3 and 4) in a dose-dependent manner (Figure 2C).

### Effects of artesunate with or without leu or 3-MA on hypoxia-induced primary hippocampal neurons in rats

**Figure 3:**
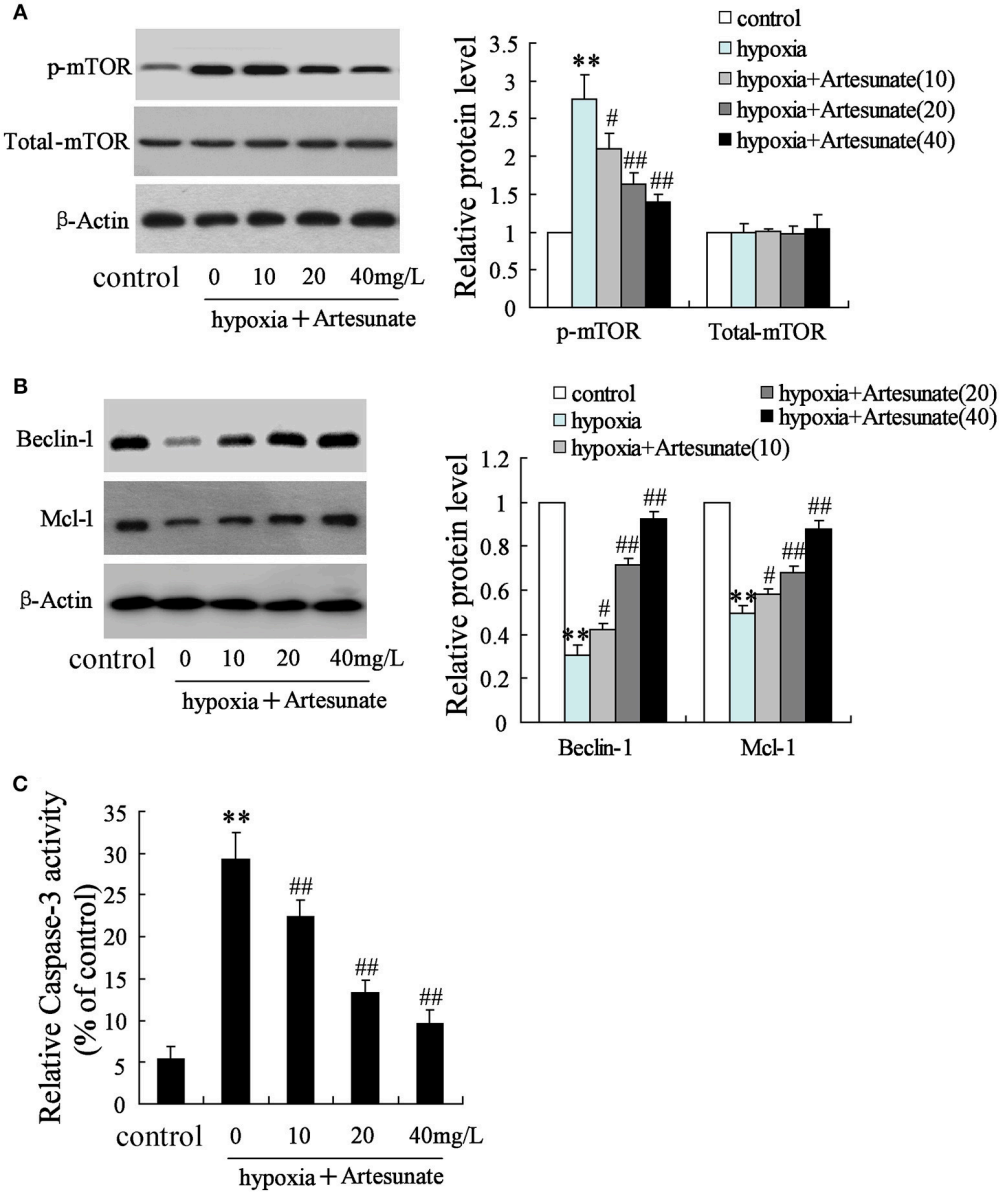
Effects of artesunate with the presence/absence of LEU or 3-MA on ischemic neuron cells. Hypoxia treatment increased the expression of p-mTOR **(A)**, decreased the expression of Beclin-1 and Mcl-1 **(B)**, and promoted cell apoptosis **(C)**, while artesunate supplementation reversed the effects in a dose-dependent manner. **P < 0.01 vs. control, ^*##*^*P* < 0.01 vs. hypoxia.

The rats' primary hippocampal neurons were divided into 4 groups and individually pretreated with 0, 10, 20, and 40 mg/L artesunate for 2 h. Afterward, the cells were treated with oxygen-glucose deprivation for 24 h. It was found that hypoxia prominently increased the expression of p-mTOR but not total-mTOR (Figure 3A). While hypoxia decreased the expressions of Beclin-1 and Mcl-1 (Figure 3B) and increased cell apoptosis (Figure 3C), all these effects were abolished by artesunate in a dose-dependent manner.

**Figure 4:**
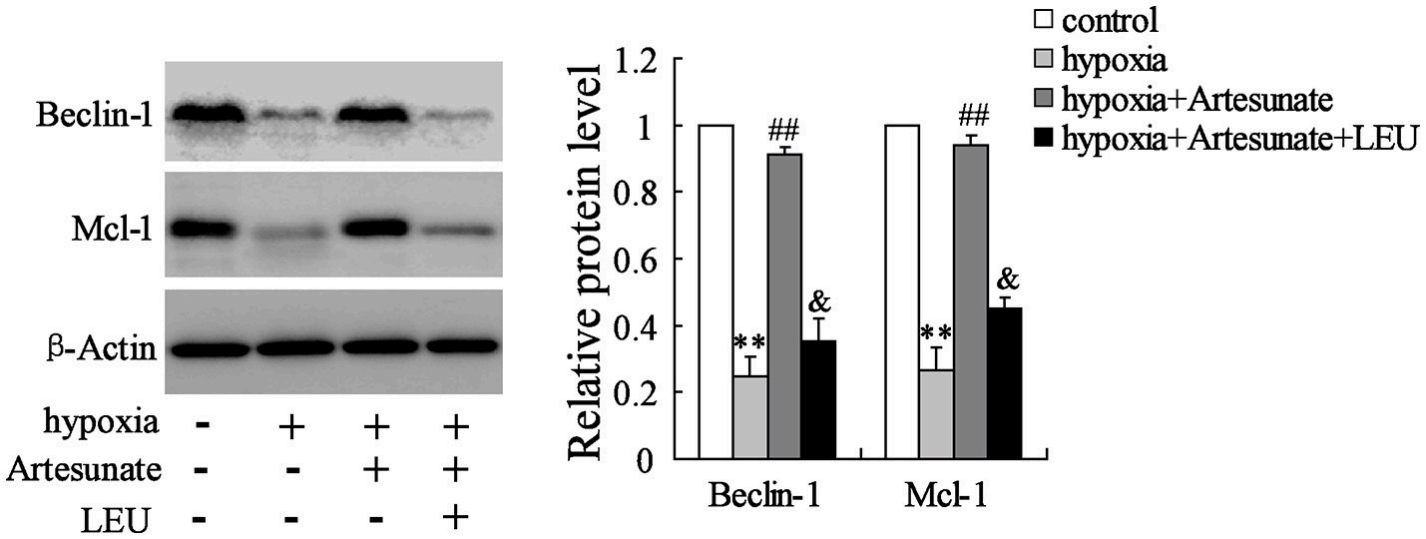
Hypoxia treatment significantly decreased the expression of Beclin-1 and Mcl-1, and the effects were reversed by artesunate supplementation; however, LEU administration abolished the effects induced by artesunate. **P < 0.01 vs. control, ^##^*P* < 0.01 vs. hypoxia, ^&^*P* < 0.05 vs. hypoxia+artesunate.

The primary hippocampal neurons of the rats were pretreated with the presence or absence of artesunate (40 mg/L) and LEU (5 mmol/L) for 2 h, then treated with oxygen-glucose deprivation for 24 h. Figure 4 demonstrates that hypoxia supplementation significantly decreases the expressions of Beclin-1 and Mcl-1 and artesunate administration seems to reverse its effects, while LEU abolishes its effects.

**Figure 5:**
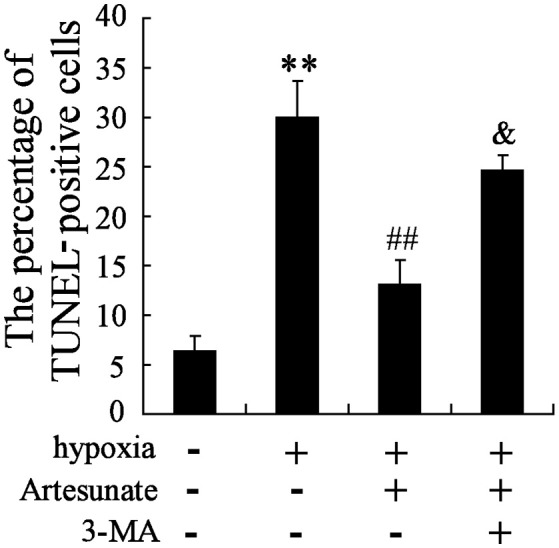
Hypoxia treatment significantly promoted cell apoptosis, and artesunate supplementation reversed the results, whereas 3-MA abolished the effects induced by artesunate (E). **P < 0.01 vs. control, ^##^*P* < 0.01 vs. hypoxia, ^&^*P* < 0.01 vs. hypoxia+artesunate (40 mg/L).

The hippocampal neurons were pretreated with the presence or absence of artesunate (40 mg/L) and 3-MA (50 mmol/L) for 2 h, then treated with oxygen-glucose deprivation for 24 h. The results revealed that hypoxia stimulation significantly increases cell apoptosis, while this effect is reversed by rapamycin; however, 3-MA supplementation abolishes the effects induced by artesunate (Figure 5).

### Effects of rapamycin and 3-MA on hypoxia-induced primary hippocampal neurons in rats

**Figure 6:**
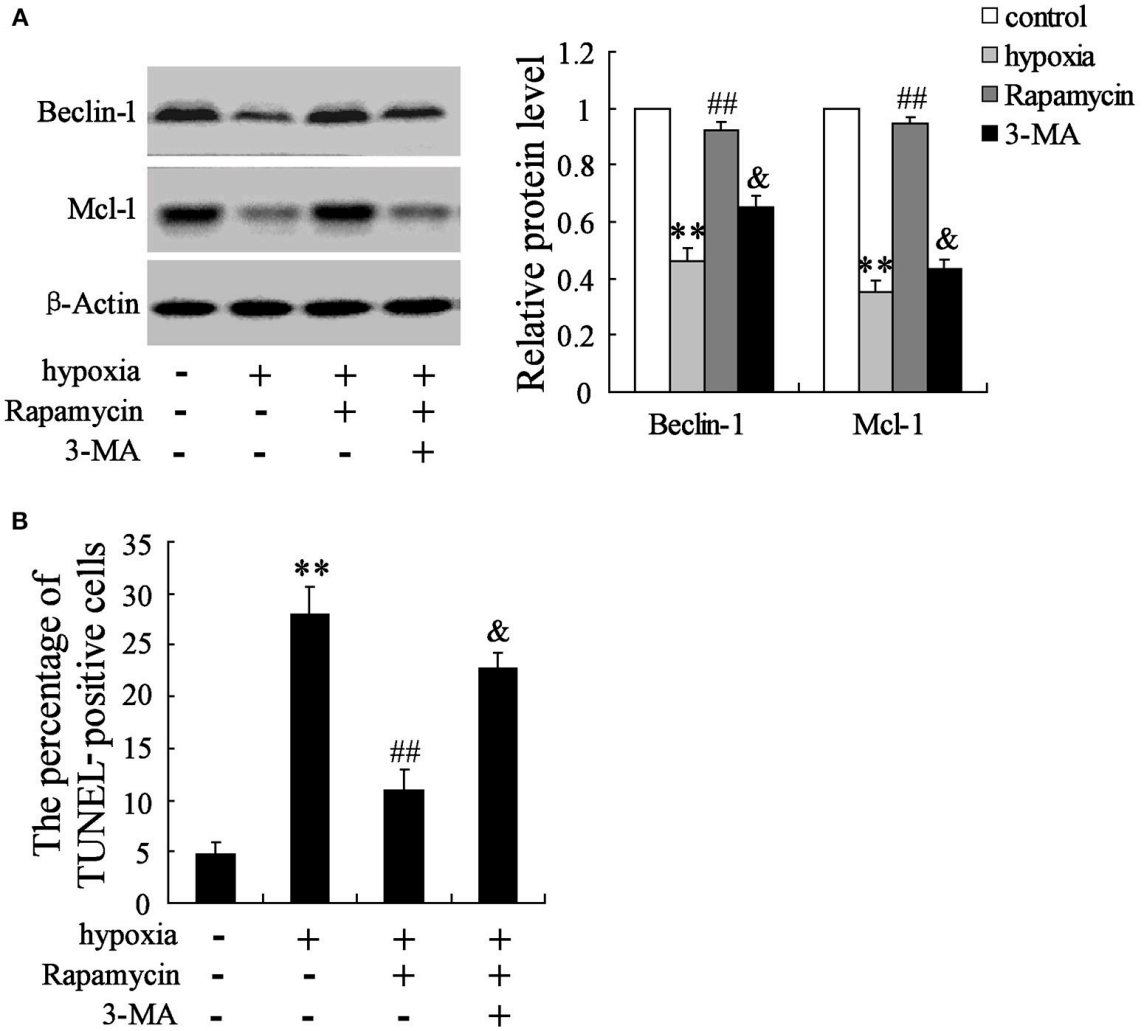
Interaction effects of rapamycin and 3-MA on neuron cells. Hypoxia treatment significantly decreased the expression of Beclin-1 and Mcl-1 **(A)**, but promoted cell death **(B)**. Rapamycin supplementation reversed the effects of hypoxia, while 3-MA administration abolished the effects induced by rapamycin. **P < 0.01 vs. control, ^##^*P* < 0.01 vs. hypoxia, ^&^*P* < 0.01 vs. hypoxia+artesunate (40 mg/L).

The hippocampal neurons were pretreated with the presence or absence of the p-mTOR inhibitor rapamycin (100 mmol/L) or the autophagy inhibitor 3-MA (50 mmol/L) for 2 h, then treated with oxygen-glucose deprivation for 24 h. As is presented in Figure 3, hypoxia stimulation significantly decreases the expressions of Beclin-1 and Mcl-1, but increases cell apoptosis. Rapamycin seems to reverse its effects, while 3-MA abolishes the effects induced by rapamycin (Figures 6A,B).

### *In vivo* examination of the effects of MCAO, artesunate and 3-MA

**Figure 7:**
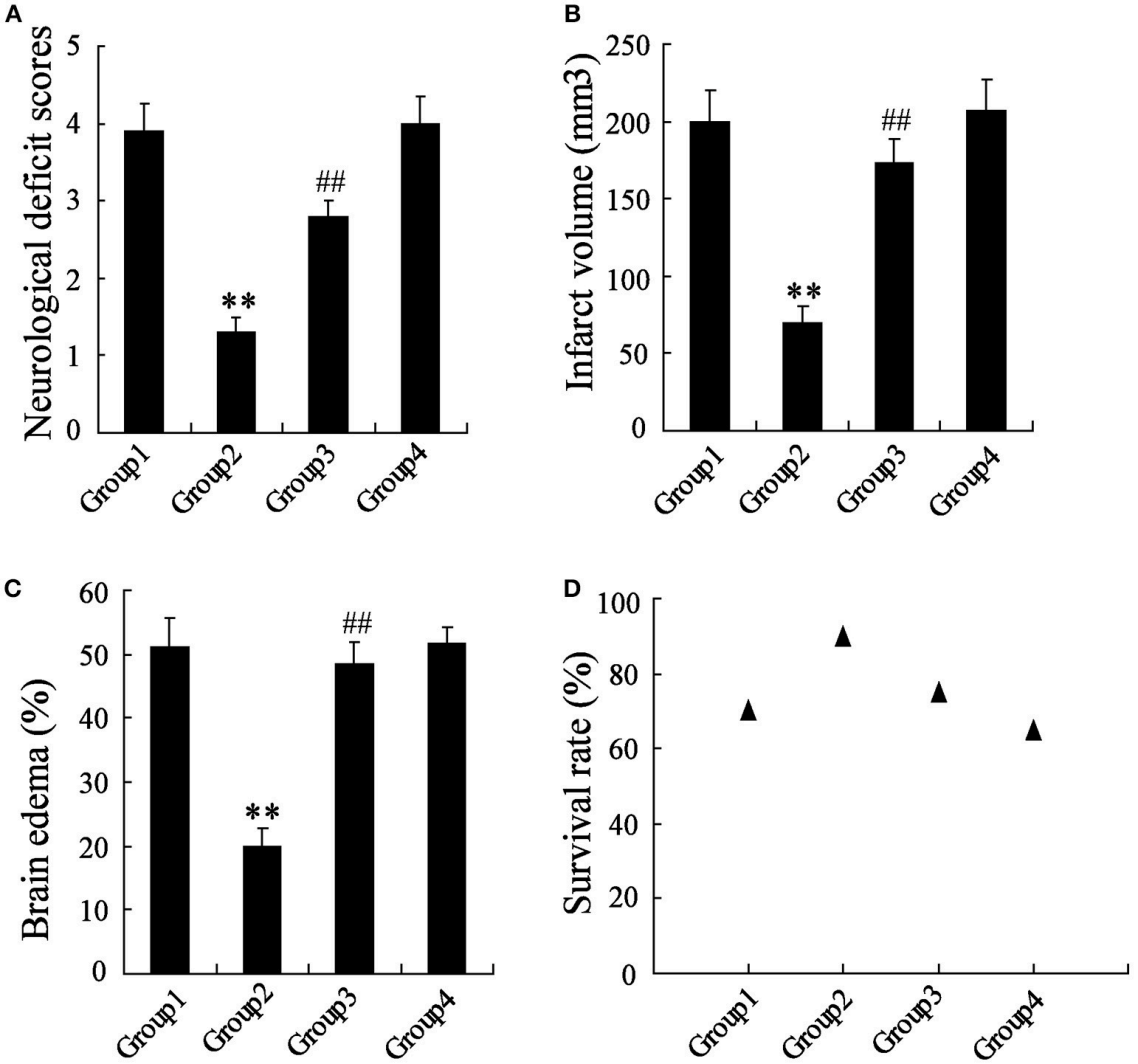
*In vivo* to verify the effects of artesunate and 3-MA on brain injury. The neurological deficit score **(A)**, infarct volume **(B)**, brain edema **(C)**, and survival rate **(D)** were significantly lower in MCAO rats that were supplied with artesunate, while 3-MA supplementation reversed the results in a dose-dependent manner. **P < 0.01 vs. Group 1, ^##^*P* < 0.01 vs. Group 2.

The MCAO rats were randomly divided into 4 groups, including Group 1: MCAO+PBS, Group 2: MCAO+artesunate (60 mg/kg), Group 3: MCAO+artesunate (60 mg/kg) +3-MA (30 mg/kg), and Group 4: MCAO+3-MA (30 mg/kg). After 24 h, the mortality rates of Groups 1, 2, 3, and 4 were 30, 10, 25, and 35% respectively (the data are not shown in the article). The indices of the live rats were detected, and the results revealed that artesunate supplementation decreases neurological deficit score (Figure 7A), infarct volume (Figure 7B), brain edema (Figure 7C), and survival rate (Figure 7D) in a dose-dependent manner, while the effects are abolished by 3-MA supplementation.

## Discussion

This study exhibited the effects of artesunate as a protective agent in ischemic cerebral infarction in rats. It was found that artesunate protects the brain from MCAO-induced cerebral infarction, based on the dose. Upon the analysis of artesunate *in vivo* experiments, the results revealed that artesunate protects the brain against ischemic infarction.

Mounting studies have reported the current efforts to improve neurorehabilitation. For example, Human induced pluripotent stem cells has been proven to improve the recovery of stroke (22). Another study revealed that Stem cell protected stroke of preclinical models is associated with aging (23). However, the therapy of hypoxia-ischemia induces brain injury was still need further exploration. Hypoxia-ischemia induces brain injury by impairing oxygen supplements, which leads to a variety of biochemical reactions in the body and disturbed normal physiological processes (24). Hypoxia-ischemia can have enormously severe symptoms; for example, it affects oxidative stress and nitrification stress in newborn rats' brains (25), and gestational and perinatal inflammation is associated with hypoxia-ischemia (26). Moreover, many studies have proven that cell death and autophagy induced by hypoxia-ischemia in the brain are an important neuron injury in patients (27–29). In this study, hypoxia-ischemia was performed to obtain brain infarction cells, which were essential to the study. Previous studies have also declared that hypoxia-ischemia can successfully induce brain infarction cells. For example, Qu et al. found that hypoxia-ischemia induces severe neuronal apoptosis *in vitro* experiments (30), and Savard et al. proved that IL-1β and MMP-9 are mediated in neuronal self-injury in hypoxia-ischemia induced neuron cells (31). All of these observations suggest that hypoxia-ischemia is crucial in brain infarction.

The mammalian target of rapamycin (mTOR) is part of the mTOR Complex 1, with the capacity to regulate cell growth and autophagy. The activation of mTOR is related to many diseases such as cancer, cardiovascular diseases, neurodegenerative diseases as well as brain diseases (32, 33); at the same time, inhibition of mTOR induces autophagy. The expression of phosphorylated mTOR (p-mTOR) is always used to measure the activation of mTOR. From this study, it was found that hypoxia-ischemia stimulation promoted the expression of p-mTOR, while artesunate supplementation impaired the expression. However, other studies provided key inhibitors of mTOR; for example, rapamycin was recognized as an inhibitor of mTOR and affects cell cycle arrest, ribosome biogenesis and autophagy (34).

In this study, Beclin-1, Mcl-1, and caspase-3 were used to assess cell apoptosis. As was presented, the expressions of Beclin-1 and Mcl-1 were significantly decreased in hypoxia-ischemia induced cells, while the expressions of p-mTOR and caspase-3 was significantly increased, which suggested that hypoxia-ischemia stimulation significantly increases cell death and reduces autophagy. Additionally, artesunate supplementation reverses the effects, indicating the protective role of artesunate on neuron cells.

To explore the effects of mTOR on autophagy, p-mTOR agonist leucine (LEU), autophagy inhibitor 3-MA, and p-mTOR inhibitor rapamycin were used individually to observe their effects on cell apoptosis and autophagy. It was found that the protective effect of artesunate on ischemic brain infarction is reversed by both LEU and 3-MA. Moreover, rapamycin also exhibits a protectiveness of neuron cells against ischemia-induced brain infarction, while its effects are abolished by 3-MA. To verify whether the results obtained *in vitro* experiments were acceptable, a *vivo* experiment with MCAO rats was then performed, and the results revealed that ameliorated injury of brain infarction, while its effects were reversed by 3-MA.

## Conclusion

This study suggests that hypoxia-induced brain infarctions significantly increase the expression of p-mTOR, Artesunate administration seems to protect the brain against infarction by decreasing the expression of mTOR, while the protectiveness is reversed by LEU and 3-MA. Additionally, rapamycin seems to reverse the effects of hypoxia, while 3-MA abolishes the effects of rapamycin. This study suggests that p-mTOR acts as a potential biomarker of brain infarctions, and artesunate provides a potential role for the therapy of ischemic brain infarctions.

## Consent for publication

The study was undertaken with the consent of the First Affiliated Hospital of Harbin Medical University.

## Author contributions

MS conceived and designed the study and drafted the manuscript. YS collected the data. HS and DM analyzed the data. WH interpreted the data. XQ participated in the study and helped to draft the manuscript. All authors read and approved the final manuscript.

### Conflict of interest statement

The authors declare that the research was conducted in the absence of any commercial or financial relationships that could be construed as a potential conflict of interest.
